# Modulation of Blood Coagulation and Hematological Parameters by *Crassocephalum crepidioides* Leaf Methanol Extract and Fractions in STZ-Induced Diabetes in the Rat

**DOI:** 10.1155/2020/1036364

**Published:** 2020-05-15

**Authors:** Opeyemi O. Ayodele, Funmilayo D. Onajobi, Omolaja R. Osoniyi

**Affiliations:** ^1^Babcock University, Ilishan, Nigeria; ^2^Department of Biological Science, College of Basic and Applied Sciences, Mountain Top University, Makogi Oba, Nigeria; ^3^Department of Biochemistry and Molecular Biology, Obafemi Awolowo University, Ife, Nigeria

## Abstract

Diabetes affects the homeostasis of the circulatory system. *Crassocephalum crepidioides* Benth S. Moore (Asteraceae) is an edible plant locally used in the treatment of wounds, stomach ulcer, and skin-related conditions in Africa and some other parts of the world. This study investigated the effects of *C. crepidioides* leaf methanol extract and fractions on blood coagulation profile of diabetic Wistar rats. The effect of 100 mg/kg body weight of the methanol extract and partitioned fractions of *C. crepidioides* on blood coagulation profile of STZ-induced diabetic rats were initially evaluated, while graded concentrations (50–200 mg/kg body weight) of the aqueous and hexane fractions were further tested in diabetic rats against standard drugs aspirin (anticoagulant) and metformin (antidiabetic). Rats were allocated into groups (*n* = 6) and administration was done orally, once daily for 2 weeks. The methanol extract and fractions of *C. crepidioides* at concentrations of 50, 100, and 200 mg/kg significantly prolonged the bleeding (58–200%), clotting (65–133%), prothrombin (176–441%), and activated partial thromboplastin (209–518%) times in diabetic rats compared to the control rats (LD_50_ ≥ 5000 mg/kg). Highest prolongation effects were recorded in the diabetic group treated with 100 mg/kg body weight of the hexane fraction. Plasma calcium concentration and platelet counts of *C. crepidioides* treated diabetic rats were significantly (*P* < 0.05) reduced compared to diabetic control rats, while the red blood cells (RBC), hemoglobin concentration, and packed cell volume (PCV) were significantly increased. This study showed that *C. crepidioides* possess anticoagulant and antianemic activities. The leaves can thus be a potential source of novel anticoagulant and nutraceutical for management of the thrombotic disorder in diabetes and other diseased states.

## 1. Introduction

Blood coagulation involves a cascade of reactions that minimize or staunch blood flow to maintain balance within the vascular system. These reactions include spontaneous vasoconstriction, aggregation of platelets, blood clotting, and fibrinolysis (clot dissolution) [1]. The process is rapid and efficient and requires regulation. This is because a shift in the balance between blood coagulation and inhibition of coagulation to favor either pro- or anticoagulation may result in life-threatening thromboembolism or hemorrhage (spontaneous bleeding) [2]. Control of this process under many clinical situations requires drug interventions that aim at preventing tissue damage caused by reduced blood flow that occurs when the coagulation process blocks the blood supply to a tissue area or an organ [1].

Diabetes mellitus and its complications is a potentially morbid condition characterized by hyperglycemia, and about 80% of people with diabetes mellitus die from thrombosis arising from enhanced activation of platelets and clotting factors [3, 4]. In the diabetic state, there is an impairment of the thrombohemorrhagic balance that exists in the blood flow of a healthy individual. This makes diabetic patients be susceptible to thromboembolic complications [5], atherosclerosis, and increased plaque rupture [6, 7]. These, in turn, may lead to aggravation of the diseased state. Erythrocyte (RBC) aggregation and decreased deformability predominate among the hematological abnormalities reported in diabetes. The structures and architecture of platelets, erythrocytes, and fibrin networks have been reported to be of importance in the pathogenesis of cardiovascular complications in diabetes mellitus [8, 9].

*Crassocephalum crepidioides* Benth S. Moore (Asteraceae), commonly called fireweed or Redflower ragleaf is an annual edible plant that is widespread in tropical and subtropical regions [10, 11]. It is locally eaten as vegetables in soups and salads and used in the treatment of fresh cuts, wounds, boils, stomach problems, and skin ailments [12–14]. *C. crepidioides* has been recently reported to have antidiabetic activity [11]. Other reported activities include antimicrobial [15], antihelminthic [14], anti-inflammatory [16], cancer chemopreventive [17], antioxidant [18], free radical scavenging, and hepatoprotective actions [19]. Given the reported antidiabetic activity of this plant, it is reasonable to evaluate its effect on blood coagulation in a diabetic model. This study, therefore, aims at investigating the effects of *C. crepidioides* leaf extract and solvent fractions on blood coagulation parameters of diabetic rats.

## 2. Materials and Methods

### 2.1. Plant Materials

*Crasssocephalum crepidioides* was locally obtained during the rainy season (July–September 2017) from farms in Ilisan-Remo, Ogun State (Latitude 6.9°N, longitude 3.7°E), Nigeria. The plant sample was identified by Mr. G. A. Ademoriyo (Botanist) at the IFE herbarium, Obafemi Awolowo University, Ile-Ife, Osun State, Nigeria. A voucher specimen was deposited with the voucher specimen registration No: IFE 17634.

### 2.2. Extraction

*C. crepidioides* leaves were oven-dried at 40°C and ground into powder using an electric blender and stored in the refrigerator at 4°C. The dried, ground sample (600 g) was soaked with 8 volumes of 70% methanol (3360 mL methanol + 1440 mL distilled H_2_O) for 48 h at room temperature accompanied by intermittent shaking [20]. After 48 h, the suspension was filtered through a fine muslin cloth and then through a No. 1 Whatman filter paper. The solvent from the crude extract was removed at a temperature of 40°C, under reduced pressure in a rotary evaporator at 40°C, then dried to completion in a hot-air oven at 40°C and stored in the refrigerator at 4°C until use. The yield of the crude extract was 15.25%. The methanol (crude) extract (85g) was then subjected to solvent partitioning using hexane, ethyl acetate, and butanol successively in order of increased polarity [21]. The residual portion after obtaining the butanol fraction was the aqueous fraction. The fractions yield from the solvent partitioning was 11.40%, 14.12%, 26.72%, and 45.40% for hexane, ethyl acetate, butanol, and aqueous fractions, respectively.

### 2.3. Drugs and Chemicals

All chemicals and drugs used were of analytical grade. Chemicals and solvents were purchased from Sigma Aldrich Chemical Co. (St. Louis, MO, USA), while streptozotocin was purchased from Santa Cruz Biotechnology, Inc., Dallas, TX, USA. Reagents for Prothrombin time and activated partial thromboplastin time were obtained from Diagen Diagnostic Reagents Ltd., Thames, Oxon, UK.

### 2.4. Acute Toxicity Study

Acute toxicity of the plant fractions was determined according to Lorke's method [22] as reported by Elufioye and Onoja [23], with a slight modification. 12 female (Nulliparous) Wistar albino rats weighing 120–130 g were used each for the crude extract and fraction. The rats were acclimatized for 2 weeks before the commencement of study. The experiment was carried out in two phases; in phase 1, nine healthy rats were allocated into three groups of three animals each. Food was withdrawn 12 h prior to the experiment, and each group of animals was administered with oral doses (10, 100, and 1000 mg/kg) of *C. crepidioides* extract. The rats were observed for 24 h to monitor their behavior and record any mortality. In phase 2, three rats were administered higher oral doses (1600, 2900, and 5000 mg/kg) of plant extract, respectively, and then observed for 24 h for change in behavior and mortality. The third group of three animals served as controls. All animals were further observed for 14 days, weighing them weekly.

### 2.5. Experimental Animals for the Study

Wistar albino rats (male) weighing 150–200 g were obtained from the animal breeding facility of Ladoke Akintola University, Ogbomoso, Oyo State, Nigeria and kept in cages at the experimental animal facility of Babcock University where they were maintained under 12 h light/dark cycle at room temperature. The animals were acclimatized for 2 weeks, fed with standard rat diet and water *ad libitum*.

Animal care and handling, as well as experimental protocols, were duly approved by the Babcock University Health Research Ethics Committee (BUHREC) with the Certificate No. BU/BUHREC436/17. In accordance with the National Institute of Health Guidelines for the Care and Use of Laboratory Animals (National Research Council (US) committee update, 8^th^ edition, 2011) and Institute for Laboratory Animal Research (ILAR), 2011 guidelines, animal suffering was minimized as much as possible and an optimum number of rats were used.

### 2.6. Induction of Diabetes

The rats were fasted overnight, and diabetes was induced in them by single intraperitoneal injection of streptozotocin (55 mg/kg body weight) in citrate buffer (pH 4.5). Injected rats were returned to their cages and provided with 5% glucose solution for the next 12 h to overcome STZ-induced hypoglycemia. After 72 h of STZ administration, the rats were fasted overnight and fasting blood glucose (FBC) was tested using blood samples from the tail vein of the rats, with a glucometer (Accu-Chek, Roche Diagnostics (GmbH, Germany and Accu-Chek test strips). Rats with sustained (after 72 h) fasting blood glucose levels >200 mg/dL were regarded as diabetic and were used for the study.

### 2.7. Experimental Protocol

The experiment was in two phases: the methanol extract and fractions (hexane, ethyl acetate, butanol and aqueous) at concentration of 100 mg/kg body weight were initially tested on diabetic rats. Animals were assigned into 8 groups with each group receiving daily oral treatment as follows:

  Group 1: Normal control + 1 mL Phosphate buffered saline (PBS)
  Group 2: Diabetic control + 1 mL PBS
  Group 3: Diabetic + hexane fraction of *C. crepidioides.*
  Group 4: Diabetic + butanol fraction of *C. crepidioides.*
  Group 5: Diabetic + aqueous fraction of *C. crepidioides.*
  Group 6: Diabetic + ethyl acetate fraction of *C. crepidioides.*
  Group 7: Diabetic + methanol (crude) extract of *C. crepidioides*
  Group 8: Diabetic + 100 mg/kg Metformin [24]

The fractions and Metformin were suspended in PBS.

In phase 2, graded concentrations (50–200 mg/kg body weight) of the aqueous and hexane fractions (the two most active fractions from phase 1) were further tested against standard drugs aspirin (anticoagulant) and metformin (antidiabetic) in a new set of rats. The rats were randomly allocated into groups of 6 rats each and assigned daily oral treatments as follows:

  Group 1: Normal control (given 1 mL PBS)
  Group 2: Normal rats given aspirin dissolved in PBS (75 mg/kg body weight) as standard anticoagulant [25]
  Group 3: Diabetic control (given 1 mL PBS)
  Group 4: Diabetic rats given aspirin dissolved in PBS (75 mg/kg body weight) [24]
  Group 5–7: Diabetic rats given the hexane fraction of *C. crepidioides* suspended in PBS (50, 100, and 200 mg/kg, respectively)
  Group 8–10: Diabetic rats given the aqueous fraction of *C. crepidioides* suspended in PBS (50,100 & 200 mg/kg respectively)
  Group 11: diabetic rats given metformin 100 mg/kg body weight [24]

The animals were kept under observation and oral administration of plant extract was done daily for 2 weeks after which blood samples were collected from the eyes by ocular puncture, transferred into centrifuge tubes containing 3.2% sodium citrate solution (1 part of trisodium citrate solution: 9 parts of blood) and centrifuged at 2500 g for 15 min to obtain pure platelet plasma (PPP) for PT and aPTT assays.

### 2.8. Clotting Time (CT) Measurement

This was carried out using Ivy's method as reported by Ibu and Adeniyi [26]. Each rat's tail was cut, and a drop of blood from the cut was placed on a clean glass slide while simultaneously starting a stopwatch. A pin was passed across the blood at an interval of 15 sec until threads of fibrin are noticed. The watch was stopped immediately, and the time recorded as the clotting time.

### 2.9. Bleeding Time (BT) Measurement

This was done according to the method described by Shrivastava and Das [27]. A cut was made on each rat's tail at 1–2 cm proximal from the end while simultaneously starting a stopwatch. At intervals of 15 sec, blood spots were made with the bleeding tail on a blotting paper until the bleeding stopped. The time taken for the bleeding to stop was recorded as the bleeding time.

### 2.10. Determination of Prothrombin Time (PT)

PT was determined following the PT reagent (Diagen calcium brain thromboplastin) manufacturer's instruction according to the method of Brown [28] with a slight modification. Calcium Rabbit Brain Thromboplastin reagent (0.2 mL) was measured in a clotting tube placed in a water bath at 37°C and incubated for 1 to 2 min to reach 37°C. Plasma (0.1 mL) was then added and a stopwatch started. The tube was slightly tilted at regular intervals (returning to the water bath between tilting) until the formation of a clot was observed. The watch was stopped, and the time was recorded.

### 2.11. Determination of Activated Partial Thromboplastin Time (aPTT)

This was done following the aPTT reagent (Diagen kaolin platelet substitute mixture) manufacturer's instruction. The reagent was reconstituted with 5 mL distilled water, and 0.2 mL of Kaolin platelet substitute mixture was measured into a clotting tube in a water bath at 37°C and incubated for 1–2 min. After that, 0.1 mL of test plasma was added, and the tube gently tilted at intervals for exactly 2 min. Then, 0.1 mL of 0.025 M calcium chloride (preincubated at 37°C) was added, while simultaneously starting a stopwatch. Tilting of the tube at regular intervals continued until clot formation was observed. The watch was instantly stopped, and the time was recorded.

### 2.12. Determination of Hematological Parameters and Calcium Concentration

The hematological profile of the whole blood of experimental animals was done using a Swelab automatic Autocounter, AC970EO+ (Boule Medicals, Sweden). Blood samples were collected into EDTA bottles, gently mixed by reversing about 10 times and left to rest at room temperature for about 15 min prior to analysis to enable the cells to stabilize.

Plasma calcium concentrations of experimental rats were determined by a colorimetric method (O-cresolphthalein complexone, without deproteinization) using assay reagents from Randox Laboratories Ltd., UK.

### 2.13. Phytochemical Characterization

The phytochemical characterization of the hexane fraction which elicited the greatest activity was done using Gas Chromatography-Mass Spectroscopy (GC-MS).

### 2.14. Statistical Analysis

The results were expressed as the arithmetic mean plus or minus standard error of mean (SEM). Data were statistically analyzed by one-way analysis of variance (ANOVA) followed by Tukey's multiple comparisons using GraphPad Prism 7.0 for Windows (GraphPad Prism Software, San Diego, CA, USA). *P* values less than 0.05 (*P* < 0.05) were considered statistically significant.

## 3. Results

### 3.1. Acute Toxicity

Administration of single doses at 10, 100, and 1000 mg/kg (in phase I) and 1600, 2900, and 5000 mg/kg (in phase II) by oral gavage failed to produce any adverse effect after 24 h in the experimental rats. Further observation of the rats for 14 days showed no mortality or abnormal behavior in any of the treatment groups. This indicates that *C. crepidioides* leaf extract and fraction is safe up to 5000 mg/kg body weight (LD_50_ ≥ 5000 mg/kg).

### 3.2. Effect of *C. crepidioides* Leaf Extract and Fractions on Coagulation Profile of Diabetic Rats

**Figure 1:**
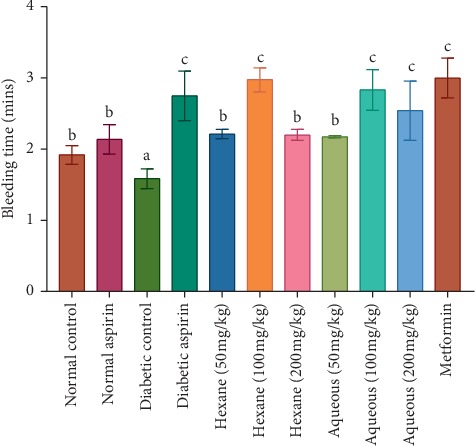
Bleeding time in diabetic rats treated with different concentrations of aqueous and hexane fractions of *C. crepidioides* b and c are significantly higher than a (*P* < 0.05). c is significantly higher than b at *P* < 0.05; *n* = 4.

**Figure 2:**
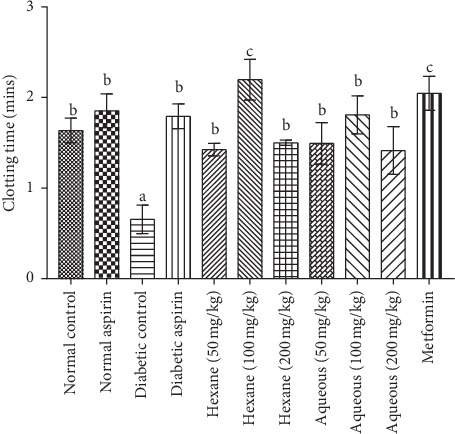
Clotting time in diabetic rats treated with different concentrations of aqueous and hexane fractions of C crepidioides b is significantly higher than a (*P* < 0.01). c is significantly higher than a (*P* < 0.001); *n* = 4.

**Figure 3:**
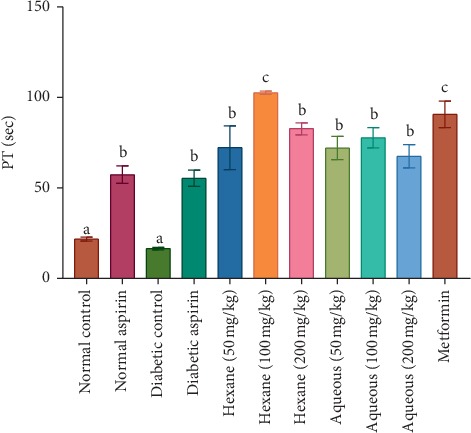
Prothrombin time (PT) in diabetic rat treated with different concentrations of aqueous and hexane fractions of *C. crepidioides*. Bars with different letters are significantly different (*n* = 4); b is significantly higher than a (*P* < 0.001). c is significantly higher than a (*P* < 0.0001).

**Figure 4:**
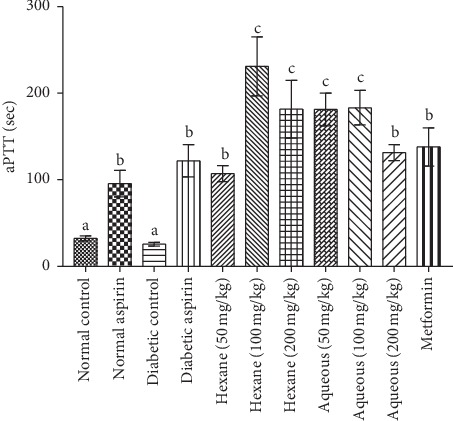
Activated partial thromboplastin time (aPTT) in diabetic rats treated with different concentrations of aqueous and hexane fractions of *C. crepidioides*. Bars with different letters are significantly different (*n* = 4); b is significantly higher than a at *P* < 0.001 c is significantly higher than a at *P* < 0.0001. b is significantly lower than c at *P* < 0.01.

The methanol extract and fractions at a concentration of 100 mg/kg body weight significantly increased the tested coagulation parameters in experimental animals with values higher than the control groups (Table 1). The BT and CT were significantly (*P* < 0.05) shorter in the diabetic control group compared to the normal rats. Although the PT and aPTT were shorter in diabetic control rats, the differences were not significant compared with normal control rats (Figures 1–4). Varying concentrations of the tested fractions of *C. crepidioides* leaf administered to diabetic rats caused significant prolongations of blood coagulation parameters tested compared to the normal and diabetic control groups. The standard drugs aspirin (anticoagulant) and metformin (antidiabetic) also elicited similar effects. The hexane fraction at 100 mg/kg body weight showed the greatest prolongation of coagulation profile of diabetic rats with values of 2.59 min, 2.13 min, 103 sec, and 232 sec recorded for bleeding time, clotting time, prothrombin time, and activated partial thromboplastin time, respectively. The results of the coagulation profile showed that the diabetic state in the rats increased blood coagulation as shown by a reduction in the times observed for the blood coagulation parameters. This was effectively countered by the anticoagulant action of *C. crepidioides* leaf extract and fractions.

Results are the mean ± SE values of duplicate determinations (*n* = 4). Mean values followed by different letters are significantly (*P* < 0.05) different while those with the same alphabet within the column are not significantly (*P* > 0.05) different. *Keys*: BT: Bleeding time, CT: Clotting time, PT: Prothrombin time, aPTT: Activated partial thromboplastin time.

### 3.3. Effect of *C. crepidioides* Leaf Fractions on Hematological Parameters and Calcium Concentration in Diabetic Rats

The results (Table 2) showed a significant decrease in red blood cell count (RBC), hemoglobin concentrations (HGB), packed cell volume (PCV), and calcium concentration of diabetic control group compared with the normal control group thus indicating the possible presence of anemia in the diabetic condition. The white blood cells (WBC) were significantly increased in the diabetic control group compared to the normal rats, while there was no significant difference in platelets count (PLT).

The RBC was significantly increased in diabetic rats given 50 and 100 mg/kg body weight of the aqueous and hexane fractions, and the standard drugs (aspirin and metformin) compared to the diabetic control, while no significant difference was recorded between diabetic rats given 200 mg/kg body weight of the two fractions and the diabetic control group. The PCV and HGB were significantly increased in diabetic rats at all tested concentrations of the aqueous and hexane fractions as well as with metformin. PLT was significantly reduced in diabetic groups given different concentrations of the aqueous and hexane fractions compared with the diabetic control group. Significant reduction of platelet count compared with the diabetic control was also recorded in diabetic rats given anticoagulant drug aspirin. However, the least count of 207.33 × 10^9^/L was recorded in diabetic rats given 100 mg/kg hexane fraction, making the hexane fraction at 100 mg/kg the most effective for platelet reducing activity.

Calcium concentrations were significantly (*P* < 0.001) lower in diabetic rats treated with all concentrations of aqueous fraction and 50 mg/kg of hexane fraction, while there were no significant differences in diabetic groups treated with 100 and 200 mg/kg of hexane fractions compared with the diabetic control group. Plasma calcium-lowering effect was also observed with metformin, while there was no difference with aspirin.

### 3.4. GC-MS Phytochemical Characterization

Compounds identified from the GC-MS analysis of the hexane fraction of *C. crepidioides* leaves with possible anticoagulant activities have been earlier reported [29]. Some of the identified bioactive compounds that may be responsible for observed activities in the present study include eugenol and *α*-linolenic acid (S/N 6 & 13) with antiaggregant activity; thujone (S/N 5) with antiplatelet activity; coumarin-related compounds, benzofuran and benzofuranone (S/N 4 & 7); *α*-linolenic acid (S/N 13) which possesses antiaggregant activity; n-hexadecanoic acid and its methyl ester (S/N 8&9) which are known to possess lipid-lowering activities (Table 3). 1,9-octadecadiene with no known activity, other phenolic compounds, flavonoids and some phytochemicals with some other biological activities were tentatively identified.

## 4. Discussion

Thromboembolic complications are a known factor for increased mortality in diabetic patients [32]. This is because patients suffering from diseases such as diabetes mellitus and cardiovascular disease are usually in a procoagulant state [33–35]. Thus, anticoagulant treatment plays an important role in reducing the risk of thrombosis in these diseased states. Over the past few decades, numerous anticoagulant drugs have been applied for clinical treatment; however, the low toxicity advantage of natural agents has become a great interest in anticoagulant research [34]. This study was conducted to investigate the effect of *C. crepidioides* leaf methanol extract and fractions on blood coagulation of diabetic rats as a means of exploring its potentials in the prevention and treatment of blood coagulation problems in normal and diseased states.

*C. crepidioides* is a common green vegetable eaten in southwestern Nigeria and other African countries with a reported high nutritive value [14, 36] and with no reported toxicity. The acute toxicity test carried out with the methanol (crude) extract and hexane fraction of *C. crepidioides* leaves in female Wistar albino rats showed no toxic effect up to a concentration of 5000 mg/kg indicating very low toxicity of the plant. This corroborates the plant local use as a vegetable in soup and dishes with no reported harm.

Clotting time is a qualitative measurement of factors involved in the intrinsic pathway [37]; hence, an anomaly in the factors of the intrinsic pathway will affect the CT. The bleeding time evaluates the vascular and platelet responses with hemostasis [38]. Similarly, Adewale et al. [39] reported that the leaf aqueous extract of *Crassocephalum rubens* (Juss. ex Jacq.) S. Moore, a related species of the plant, is not toxic up to a dosage of 1000 mg/kg body weight in rats.

The recorded BT and CT in diabetic control rats were shorter than those of the normal control rats. This agrees with previous reports of increased blood coagulation in the diabetic state [40, 41]. In the present study, administration of different concentrations of C. *crepidioides* methanol extract and fractions significantly increased the BT and CT in STZ-induced diabetic rats, with similar increases recorded with aspirin and metformin. Highest prolongation effects were recorded in the diabetic group treated with 100 mg/kg body weight of the hexane fraction. The same bleeding and clotting times prolongation effects were recorded with standard drugs aspirin and metformin. These suggest an anticoagulant effect of *C. crepidioides* and thus, its potential use for treatment of coagulation disorders (e.g., thromboembolism) associated with diabetes mellitus.

Aspirin and other anticoagulants have been reported to increase bleeding time in animals and humans while procoagulants have the opposite effect [38]. Aspirin is an antiplatelet drug that irreversibly blocks the production of thromboxane *A*_2_ in platelets, thus exhibiting an inhibitory effect on platelet aggregation [42]. Treatment with *C. crepidioides* methanol extract and fractions resulted in significant decreases in PLT compared with control rats. The values recorded with the plant treatment were also significantly higher than with the diabetic group given aspirin. The observed PLT decrease, coupled with increases in BT and CT, agree with reports that there is an inverse relationship between bleeding time and platelets count [38, 43].

The PT is a coagulation parameter used in evaluating the tissue factor pathway and thus the activities of the factors of the extrinsic coagulation pathway, while the aPTT determines the activities of factors involved the contact (intrinsic) and the common pathways. The PT and aPTT are standard tests for monitoring coumarin and heparin therapies, respectively [29, 44]. The observed prolongation of both the PT and aPTT in experimental rats after *C. crepidioides* administration suggests a reduction in activities or inhibition of factors V, X, II of the common coagulation pathway. Thus, a possible mechanism for *C. crepidioides* inhibition of coagulation pathway may be by direct inhibition of the common pathway where *C. crepidioides* active component(s) decreases thrombin generation and directly or indirectly inhibits factors Xa and its cofactor Va. The active component(s) of *C. crepidioides* may also act as direct thrombin inhibitors by binding to thrombin and blocking its interaction with the substrate (fibrinogen), therefore preventing fibrin formation and platelets activation. Hence, suggesting a possible presence of a protease inhibitor in *C. crepidioides* may inhibit these clotting proteins (proteases), ultimately preventing the conversion of the zymogens to active factors Xa, Va, and thrombin.

Hyperglycemia causes nonenzymatic glycation of Antithrombin (natural anticoagulant) and depressed its biological activity and also decreases the concentration of protein C [7]. This impaired function of natural anticoagulant activates clotting factors and contributes to the onset of hypercoagulability in diabetes mellitus. Another possible mechanism of *C. crepidioides* activity is the activation of the natural anticoagulation pathway in which the active component(s)/protease inhibitor in *C. crepidioides* binds to Antithrombin resulting in its activation and conversion of protein C to activated protein C (APC), which in combination with its cofactor protein S inhibits factors Va and VIIIa and thus, inactivation of factor Xa and thrombin (common pathway). This study clearly showed the anticoagulant activity of *C. crepidioides* leaf methanol extract and fractions. This suggests that the local use of the plant in cut and wound healing may be due to antibacterial and antifungal activities of some of the bioactive compounds identified in the plant.

Assessment of hematological parameters gives an indication of the deleterious effect of the diseased diabetic state. The results showed significantly decreased RBC, HGB, and PCV in diabetic control rats compared to normal rats, with accompanied increased WBC. These results agree with the report of Kumar et al. [45] that there was significantly lowered RBCs, PCV, and MCV; and significantly higher WBC in diabetic compared to nondiabetic patients in a rural tertiary center in India. Kwon and Ahn [46] similarly reported low hemoglobin concentration in diabetes mellitus. The lowered RBC in diabetic rats could be accounted for by the destruction of mature RBC leading to decreased hemoglobin concentration that is usually accompanied by lowered PCV [47]. Akindele et al. [48] and Kumar et al. [45] reported that anemia is a common pathophysiology condition associated with diabetes mellitus. These effects were mitigated by administration of *C. crepidioides* to diabetic rats confirming the antianemic activity of the plant. The significant reduction of WBC after administration of *C. crepidioides* to diabetic rats indicates the ability of the plant to protect against diabetes-induced elevation of total white blood cell counts.

Determination of plasma calcium concentration in experimental rats showed no significant difference in plasma calcium concentrations of diabetic control rats and normal rats. Treatment of diabetic rats with the aqueous fraction of the plant at all tested concentrations significantly reduced the plasma calcium concentration compared to the normal and diabetic control rats, while there was no significant difference with the hexane fraction. Rooney et al. [49] and Becerra-Tomás et al. [50] reported that elevated serum calcium is associated with abnormalities and greater risks of diabetes. The observed varied effect of the aqueous and the hexane fraction on plasma calcium concentration in experimental animals suggest that polar (aqueous) soluble compounds are mainly responsible for the plant calcium-lowering activity. In blood coagulation, calcium mediates the formation of the tenase and prothrombinase complexes through the binding of the *γ*-carboxyl amino residue of factors Xa and IXa to phospholipid surface of the platelets, thus, activating prothrombin and cleavage of fibrinogen to fibrin [51]. It also plays a role in factor Xa inhibition by protein Z-dependent protease inhibitor (ZPI) (Corral et al.) [52]. *C. crepidioides* may probably inhibit blood coagulation through its reduction of intracellular calcium thus limiting the amount of calcium available for the formation of tenase and prothrombinase complex necessary for the activation of prothrombin to thrombin, eventually preventing fibrin formation.

The GC-MS characterization of the hexane fraction of *C. crepidioides* leaf identified different bioactive compounds with various biological activities. These include phenolic compounds, alpha-linolenic acid with antiaggregant activity, benzofuranone (a coumarin-related compound) which may have anticoagulant activity, and flavonoids reported to inhibit platelet aggregation [53]. Eugenol was reported as an anticoagulant agent in *Cinnamomum cassia* Blume (Lauraceae) [54]. It has been demonstrated to inhibit platelets aggregation by inhibiting the formation of thromboxane B2 (TXB2) in platelets through inhibition of cyclooxygenase [55, 56]. Coumarin-related benzofuranone may act as Vitamin K antagonist, preventing the formation of the active form of Vitamin K dependent clotting factors II, IX, and X. The antibacterial, antifungal, and antiseptic activities of some of the identified bioactive compounds in the plant may be responsible for its ethnomedical use in wound treatment.

## 5. Conclusion

This study shows that *C. crepidioides* leaves are relatively safe, containing bioactive compounds which possess anticoagulant and antianemic activities with great potentials in the development of novel anticoagulant and antidiabetic nutraceuticals with few side effects. Therefore, the plant leaves can potentially be used in the management of thrombotic disorders in diabetes and other diseased states such as coronary heart disease and vascular disease.

## Figures and Tables

**Table 1:** Coagulation profile of diabetic rats treated with 100 mg/kg body weight of *C. crepidioides* crude extract and fractions.

Parameters group	BT (minutes)	CT (minutes)	PT (seconds)	aPTT (seconds)
Normal control	2.00 ± 0.11^b^	1.58 ± 0.14^d^	25.00 ± 2.43^h^	31.00 ± 2.92^b^
Diabetic control	1.37 ± 0.12^a^	1.48 ± 0.12^d^	17.00 ± 2.42^g^	22.00 ± 0.56^a^
Hexane	2.39 ± 0.15^b^	3.45 ± 0.15^f^	92.00 ± 8.09^k^	136.00 ± 9.39^d^
Butanol	2.17 ± 0.16^b^	2.44 ± 0.10^e^	81.00 ± 3.63^k^	74.00 ± 9.32^c^
Aqueous	4.11 ± 0.50^c^	3.48 ± 0.19^f^	66.00 ± 6.37^j^	126.00 ± 6.96^d^
Ethyl acetate	3.58 ± 0.40^c^	2.54 ± 0.13^e^	74.00 ± 5.53^k^	74.00 ± 6.91^c^
Methanol	2.29 ± 0.05^b^	2.08 ± 0.16^d^	47.00 ± 2.46^i^	68.00 ± 1.73^c^
Metformin	3.50 ± 0.22^c^	2.58 ± 0.18^e^	68.00 ± 8.77^j^	115.00 ± 10.39^d^

**Table 2:** Hematological parameters of experimental rats treated with varying concentrations of aqueous and hexane fractions.

Parameters	NC	NA	DC	DA	Hex 50	Hex 100	Hex 200	Aq. 50	Aq. 100	Aq. 200	Met
RBC (x 10^12^/L)	7.70 ± 0.01^b^	7.01 ± 0.66^b^	6.44 ± 0.10^a^	6.86 ± 0.19^b^	7.13 ± 0.12^b^	7.36 ± 0.14^b^	6.50 ± 0.40^a^	6.81 ± 0.08^b^	7.22 ± 0.37^b^	6.45 ± 0.03^a^	7.30 ± 0.13^b^
HGB (g/dl)	14.13 ± 0.09^b^	13.57 ± 0.67^b^	11.20 ± 0.31^c^	11.40 ± 0.40^c^	13.40 ± 0.23^b^	13.73 ± 0.07^b^	12.63 ± 0.23^b^	12.40 ± 0.29^b^	14.20 ± 0.69^b^	12.53 ± 0.03^b^	13.27 ± 0.19^b^
WBC (x10^9^/L)	4.00 ± 0.12^a^	3.50 ± 0.36^a^	9.17 ± 0.61^c^	3.50 ± 0.64^a^	4.80 ± 0.92^b^	4.13 ± 0.07^a^	5.47 ± 0.75^b^	6.80 ± 0.35^b^	6.70 ± 0.92^b^	4.77 ± 0.84^b^	3.90 ± 0.06^a^
PCV (%)	43.17 ± 0.49^h^	39.53 ± 2.22^g^	32.10 ± 1.13^f^	38.60 ± 1.33^g^	33.23 ± 6.68^f^	42.20 ± 0.23^h^	38.13 ± 0.78^g^	36.90 ± 0.72^g^	39.50 ± 1.16^g^	34.90 ± 0.64^g^	38.73 ± 0.09^g^
PLT (x10^9^/L)	518.00 ± 6.25^m^	516.33 ± 1.16^m^	485.67 ± 22.2^m^	333.33 ± 18.49^l^	345.67 ± 18.41^l^	207.33 ± 4.37^k^	337.67 ± 12.02^l^	264.33 ± 26.57^k^	388.33 ± 18.95^l^	265.67 ± 25.53^k^	383.67 ± 3.84^l^
Ca^2+^ (mg/dl)	8.90 ± 0.03^b^	8.40 ± 0.03^a^	8.70 ± 0.10^b^	8.30 ± 0.03^a^	8.40 ± 0.03^a^	8.90 ± 0.03^b^	8.60 ± 0.03^b^	8.50 ± 0.03^a^	8.40 ± 0.03^a^	8.40 ± 0.03^a^	8.50 ± 0.03^a^

Results are the mean ± SE values of duplicate determinations (*n* = 4). Mean values followed by different letters are significantly (*P* < 0.05) different while those with the same alphabet within the row are not significantly (*P* > 0.05) different. *Keys*: RBC: red blood cells, HGB: hemoglobin, WBC: white blood cells, PCV: packed cell volume, PLT: platelet count, NC: normal control, NA: normal aspirin, DC: diabetic control, DA: diabetic aspirin, Hex 50, 100 &200: hexane 50 mg/kg, 100 mg/kg &200 mg/kg, Aq. 50,100 &200: aqueous 50 mg/kg, 100 mg/kg &200 mg/kg, Met: metformin.

**Table 3:** Some GC—MS identified bioactive compounds of the hexane fraction of *C. crepidioides* leaf extract that may be responsible for observed activities.

S/N	Retention time (mins)	Name of compound (library ID)	Molecular formula	Peak area (%)	Reported biological activity
1	3.586	Butyrolactone	C_4_H_6_O_2_	0.98	Antimicrobial. Central nervous system depressant (CNS) and hypnotic. Anaesthetic.
2	5.449	Benzene acetaldehyde	C_8_H_8_O	1.11	Antioxidant antibacterial, anaesthetic.
3	5.568	1-methyl, 2-Pyrrolidinone	C_5_H_9_NO	2.69	Surfactant, antifungal antioxidant, antibacterial anticancer, anticonvulsant.
4	10.286	Benzofuran	C_8_H_6_O	1.43	Antidepressant, anticancer, antiviral, antifungal, antioxidant, antipsychotic, anti-inflammatory.
5	13.640	Thujone	C_10_H_16_O	0.56	Antiplatelet antibacterial, antifungal, antinociceptive, insecticidal, anthelmintic antioxidant.
6	14.180	Eugenol	C_10_H_12_O_2_	4.43	Anti-inflammatory, antiseptic, antiaggregant.
7	19.795	Benzofuranone	C_8_H_6_O	2.99	Antioxidant, anticancer
8	22.151	1,9-octadecadiene	C_18_H_34_	0.78	Not stated
9	24.816	Orcinol	C_7_H_8_O_2_	3.14	Antifungal, antimicrobial, and keratolytic.
10	26.704	Hexadecanoic acid, methyl ester	C_17_H_34_O_2_	1.48	Antioxidant, hypocholesterolemic, nematicide, Pesticide, antiandrogenic, flavor, hemolytic, 5-alpha reductase inhibitor.
11	27.250	n-Hexadecanoic acid	C_16_H_32_O_2_	1.19	Antioxidant, anti-inflammation hypocholesterolemic, nematicide Pesticide, Lubricant, antiandrogenic, flavor, hemolytic, 5-alpha reductase inhibitor.
12	28.816	7,10,13-Hexadecatrienoic acid, methyl ester	C_20_H_36_O_3_	5.74	Antibacterial, antifungal.
13	29.404	9,12,15-Octadecatrienoic acid (*α*-linolenic acid)	C_18_H_30_O_2_	4.52	Anti-inflammatory, Hypolipidemic, antiaggregant, Antileukotriene, antiprostatic, immunostimulant, vasodilator, 5-alpha reductase inhibitor.

Main activity sources*:* Duke [30, 31].

## Data Availability

All data supporting the findings of this study are included within the article. Plant materials used in this study were obtained from the IFE herbarium where voucher specimen was deposited.
